# Assessment of the Effectiveness of Early Versus Delayed Invasive Strategies in Non-ST Elevation Myocardial Infarction (NSTEMI): A Systematic Review and Meta-Analysis

**DOI:** 10.7759/cureus.109425

**Published:** 2026-05-22

**Authors:** Aishwarya Kamble, Narasimha Reddy Revunuru, Brahmaiahchari Rangachari, Viraj Bharat Patel, Imdad Ullah, Zubair Ahmed

**Affiliations:** 1 Internal Medicine, St. Mary Medical Center, Langhorne, USA; 2 Internal Medicine, Anam Chenchu (AC) Subba Reddy Government Medical College, Nellore, IND; 3 Biomedical Sciences, Kentucky College of Osteopathic Medicine, University of Pikeville, Pikeville, USA; 4 Internal Medicine, School of Medicine, D. Y. Patil University, Navi Mumbai, IND; 5 Internal Medicine, Khyber Medical College, Peshawar, PAK; 6 Internal Medicine, Richmond University Medical Center, New York, USA

**Keywords:** delayed invasive strategy, early invasive strategy, major adverse cardiovascular events (mace), nstemi, percutaneous coronary intervention (pci)

## Abstract

In recent years, early invasive strategies for non-ST-elevation myocardial infarction (NSTEMI) have been increasingly studied as a means of promptly identifying and treating culprit coronary lesions. However, the optimal timing of invasive management remains uncertain, particularly because most available evidence is derived from developed or high-resource healthcare settings. This systematic review and meta-analysis compared early vs. delayed invasive strategies in adults with NSTEMI for major adverse cardiovascular events and key secondary outcomes. A Population, Intervention, Comparison, Outcome framework guided eligibility criteria, and relevant studies were identified through PubMed/MEDLINE, Cochrane, and Embase. Preferred Reporting Items for Systematic Review and Meta-Analysis guidelines were followed, and quantifiable outcomes were analyzed using RevMan v5.4.1 (The Cochrane Collaboration, London, UK). Ten studies were included. Overall, five of 10 studies reported significant benefits favoring early or immediate invasive strategies, four found no significant benefit in adjusted or long-term analyses, and one reported increased adverse events with early intervention. Meta-analysis showed that early intervention significantly reduced all-cause mortality (risk ratio (RR) = 0.68, 95% confidence interval (CI): 0.61-0.76, p < 0.00001) and rehospitalization due to heart failure (RR = 0.41, 95% CI: 0.29-0.58, p < 0.0001). However, no significant benefit was observed for nonfatal acute myocardial infarction (RR = 0.62, 95% CI: 0.18-2.11, p = 0.45) or cardiovascular death at five years (RR = 0.99, 95% CI: 0.66-1.48, p = 0.94). Early invasive intervention within 24 hours may improve selected NSTEMI outcomes, especially mortality and heart-failure rehospitalization. However, because the included studies were predominantly from developed countries, these findings may not be directly applicable to low-resource settings where catheterization facilities, specialists, and rapid referral systems are limited. Further evidence from low- and middle-income regions is needed.

## Introduction and background

Non-ST-elevation myocardial infarction (NSTEMI) is categorized as one of the composites of acute coronary syndrome (ACS) and carries significant morbidity and mortality. Prompt diagnosis and timely intervention are necessary to avoid complications, such as arrhythmias, cardiogenic shock, and heart failure, secondary to pulmonary edema and fluid overload [1]. The median age at presentation for ACS in the United States is 68 years. The male-to-female ratio for overall incidence is 3:2. The incidence of ACS in the United States exceeds 780,000, with NSTEMI accounting for nearly 70% of those cases [2]. The mortality rate from non-ST-segment elevation acute coronary syndrome (NSTE-ACS) was double that of ST-segment elevation myocardial infarction (STEMI), according to a study [3]. The pathophysiology behind NSTEMI is a mismatch between oxygen delivery and myocardial oxygen consumption, which creates a flow-limiting setting in the cardiac muscle cells [4]. A stable plaque, vasospasm as in Prinzmetal angina, coronary embolism, or coronary arteritis are all causative factors of the flow-limiting conditions. Indirect myocardial insults, such as cardiac contusion, myocarditis, or the presence of cardiotoxic chemicals, can also cause NSTEMI [5]. Finally, the development of NSTEMI can also be triggered by disorders unrelated to the coronary arteries or myocardium, such as hypotension, hypertension, tachycardia, aortic stenosis, and pulmonary embolism, where the increased oxygen demand cannot be met.

NSTEMI is diagnosed in patients who have ACS-like symptoms and a troponin increase but no ECG abnormalities that indicate STEMI. The primary distinction between unstable angina and NSTEMI is the presence or absence of detectable troponin leak [6]. Early invasive strategies, such as angiography and revascularization, as well as antithrombotic therapies, are recommended for the therapy of NSTE-ACS. These are vital approaches with expanding strengths in the class of recommendation and level of evidence in revised guidelines [7,8]. According to a study, an early invasive strategy was found to be superior to a delayed, conservative, or selective invasive strategy in reducing death and myocardial infarction (MI) in patients with NSTEMI [9]. In a meta-analysis comparing immediate/early invasive and delayed invasive management approaches, the early invasive approach was associated with a lower risk of recurrent ischemia and mortality, while the immediate revascularization showed a clinical benefit in reducing the risk of major bleeding [10].

In recent years, the efficacy of early invasive strategies for NSTE-ACS has been increasingly reported [11,12]. However, the clinical prognosis for NSTEMI patients varies depending on the severity of the disease, which can range from mild unstable angina to fatal acute myocardial infarction (AMI) with cardiogenic shock [13-15]. The American College of Cardiology/American Heart Association recommendations provide two options for routine procedures. Invasive strategies include early (immediate) or deferred angiography. Early angiography can identify patients with left main or multivessel disease, which may require coronary artery bypass graft (CABG) or percutaneous coronary intervention (PCI) [16]. In addition, expediting intervention may reduce antithrombotic treatment use and the accompanying increased risk of bleeding. Early intervention may prevent procedure-related complications following severe antithrombotic and anti-ischemic therapy [17].

Current guideline-based management of NSTE-ACS recommends that the timing of invasive intervention should be guided by baseline risk rather than by a single universal cutoff. Very high-risk patients, such as those with hemodynamic instability, refractory chest pain, malignant arrhythmias, acute heart failure, or cardiogenic shock, are generally prioritized for an immediate invasive strategy, commonly within two hours. High-risk patients, including those with confirmed NSTEMI, dynamic ST-T changes, or elevated Global Registry of Acute Coronary Events (GRACE) risk scores, are recommended for an early invasive strategy within 24 hours, while intermediate-risk patients may undergo delayed angiography within 24-72 hours. Therefore, although the present review uses a 24-hour cutoff to define “early” intervention, this threshold should be interpreted within the broader risk-stratified framework of contemporary NSTEMI care. Some recent evidence suggests that a more immediate strategy, particularly intervention within two hours, may reduce major adverse cardiac events in selected high-risk NSTEMI patients. However, because included studies used heterogeneous timing definitions, ranging from immediate intervention to angiography within 24 or 48 hours, the 24-hour threshold was retained as the most practical and commonly reported cutoff for synthesis. This discrepancy highlights the need to clearly distinguish among "immediate," "early," and “delayed” invasive strategies when interpreting pooled outcomes and applying findings to clinical practice [13].

## Review

Methods

Eligibility Criteria

Preferred Reporting Items for Systematic Review and Meta-Analysis (PRISMA) guidelines and the "Population, Intervention, Comparison, Outcome (PICO)" scheme were utilized to generate the eligibility criteria [18]. Studies were considered eligible for inclusion if they were published between June 2010 and June 2025. The target population included adults (≥18 years) with NSTEMI diagnosed by clinical presentation ± troponin/ECG criteria. Studies were considered for inclusion if they presented qualitative data about comparative outcomes. The PICO scheme for the current study is further elaborated in the table below (Table 1).

**Table 1:** PICO framework for the literature search NSTEMI: non-ST-elevation myocardial infarction; NSTE-ACS: non-ST-segment elevation acute coronary syndrome; MACE: major adverse cardiovascular event; CV: cardiovascular; MI: myocardial infarction; QoL: quality of life Source: [18]

Element	Description
Population (P)	Adults (≥18 years) with NSTEMI diagnosed by clinical presentation ± troponin/ECG criteria. Studies with mixed NSTE-ACS populations are eligible only if NSTEMI data are reported separately or can be extracted
Intervention (I)	Early invasive strategy: coronary angiography with possible revascularization performed early after presentation. Default definition for the review: ≤24 hours from presentation/admission (but accept study-specific timing; record exact timing and perform subgroup analyses)
Comparator (C)	Delayed/late invasive strategy: coronary angiography performed after the early window (commonly >24 hours); includes study-defined delayed timing (e.g., 24-72 hours, >72 hours). Record the study definition precisely
Outcomes (O)	Primary: MACE - predefined as CV death, nonfatal MI, and nonfatal stroke (3-point MACE). Secondary: all-cause mortality, individual MACE components, recurrent MI, urgent revascularization, heart-failure hospitalization, major bleeding, length of stay, QoL, and procedure-related complications

The inclusion criteria were carefully developed after the PICO criteria were established for the systematic review. The details of the eligibility criteria are provided in Table 2.

**Table 2:** Inclusion and exclusion criteria for the review NSTEMI: non-ST-elevation myocardial infarction; STEMI: ST-elevation myocardial infarction; PCI: percutaneous coronary intervention; MACE: major adverse cardiovascular event; RCTs: randomized controlled trials

Criterion	Include	Exclude
Population	Adults (≥18) with NSTEMI. Mixed populations where NSTEMI results are separable	Studies exclusively of STEMI, unstable angina without NSTEMI, or pediatric populations
Intervention/comparator	Studies that directly compare an early invasive strategy (study-defined; preferably ≤24 hours) vs. a delayed/late invasive strategy	Studies without a timing comparison (e.g., single-arm cohorts of PCI timing), or comparing different PCI techniques without timing contrast
Outcomes	Studies reporting MACE or its components, or data enabling the extraction of primary/secondary outcomes	Studies that report only surrogate outcomes unrelated to clinical events, or provide insufficient outcome data, and authors cannot be contacted for data
Study design	RCTs, nonrandomized comparative trials, prospective/retrospective cohorts with comparator groups	Case reports, case series with <10 patients, reviews, editorials, and preclinical work
Timeframe	Published between 2010 and 2025 (inclusive)	Publications before 2010
Language	English language publications (non-English eligible if an accurate translation is available)	Untranslated non-English reports where data cannot be reliably extracted
Publication type	Peer-reviewed full-text articles, prospective trial reports, registry analyses with comparative timing	Abstracts only (unless sufficient data and author contact yield full data), gray literature with no extractable data
Outcome reporting	Studies reporting hazard ratios for time-to-event outcomes when available, or counts/risks, allowing effect estimate calculation	Studies with no extractable numeric outcome data and no author-provided access
Definitions/timing	Accept study-specific timing thresholds; require authors’ reported timing windows and record them for subgroup/sensitivity analyses	Studies that do not report the timing of the invasive strategy at all

Information Sources

We searched through several digital databases to retrieve relevant literature. Among these are Cochrane, PubMed, Embase, and Medline. Other resources, including independent journals, were also included. The information was compiled using databases as well as journals such as "JAMA Network Open," "Elsevier," and others.

Search Strategy

The search strategy was devised following the PICO scheme to retrieve pertinent data from digital databases. In the final sample, 10 studies (out of a total of n = 183) met the eligibility criteria. A search query was formulated encompassing the following keywords:

("Myocardial Infarction, Non-ST Elevation" [MeSH Terms] OR "NSTEMI" [tiab] OR "non ST elevation myocardial infarction" [tiab] OR "non-ST-elevation myocardial infarction" [tiab]) AND ("Angioplasty, Balloon, Coronary" [MeSH Terms] OR "Percutaneous Coronary Intervention" [tiab] OR "PCI" [tiab] OR "Coronary Angiography" [MeSH Terms] OR "invasive strategy"[tiab] OR "early invasive"[tiab] OR "delayed invasive"[tiab] OR "early intervention"[tiab] OR "delayed intervention" [tiab] AND ("early" [tiab] OR "delayed" [tiab] OR "timing" [tiab] OR "time to treatment" [tiab]) AND ("Randomized Controlled Trial"[pt] OR "Cohort Studies"[MeSH Terms] OR "Observational Study"[Publication Type] OR "clinical trial"[pt] OR ("comparative study" [pt]) AND ("2010/06/01" [Date-Publication]: "2025/06/01" [Date-Publication]) AND (humans [MeSH Terms] AND adult [MeSH Terms]).

Selection Process

The study methodology was developed based on a review of relevant peer-reviewed literature. Articles meeting the predefined inclusion criteria were appraised using the PICO framework to ensure methodological strength. The screening and selection process was facilitated using Rayyan.ai (Rayyan Systems Inc., Cambridge, MA), an evidence-based platform designed to streamline the review of primary and secondary sources [19]. Following this, a total of 10 studies were found to be appropriate for inclusion. Articles were excluded if they targeted a nonrelevant population, used the wrong study design, failed to measure appropriate target outcomes, or exhibited a high risk of bias. In several cases, studies were excluded for more than one of these reasons.

Data Items

**Figure 1:**
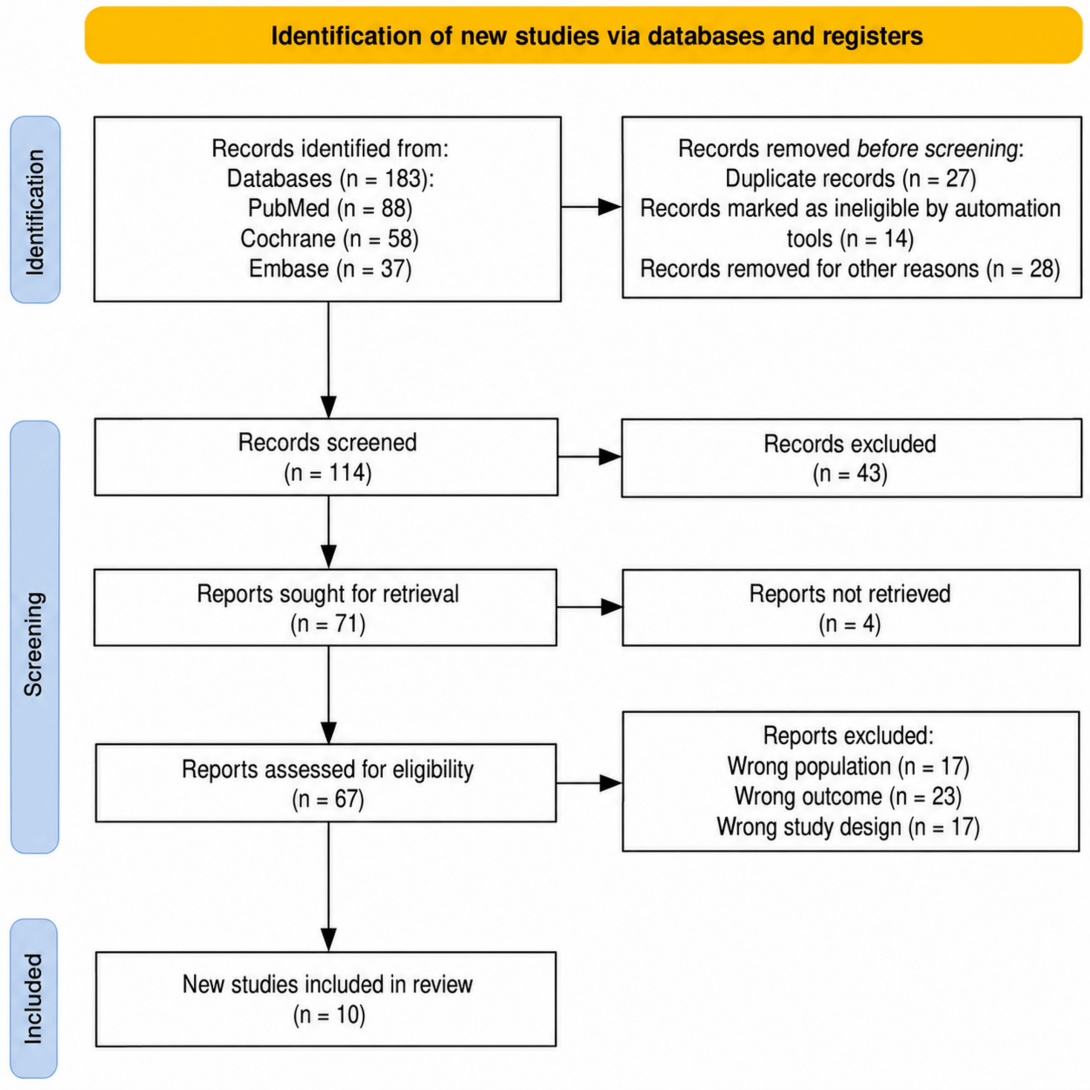
PRISMA chart for the Systematic Review PRISMA: Preferred Reporting Items for Systematic Review and Meta-Analysis

After finalizing the secondary screening process, we assessed the overall sample size (n = 10) of the selected literature. To create a PRISMA flowchart that adheres to PRISMA guidelines, we used articles from reputable journals and other sources (Figure 1) [20].

Risk-of-Bias Assessment

We conducted a comprehensive analysis of bias in each study selected for quality assessment. This required analyzing the population demographics, the characteristics of the interventions, and the region where the study was conducted. Since the studies included were randomized controlled trials (RCTs), as well as the observational cohorts, we used the Cochrane Risk of Bias (ROB) v2 tool to determine the bias in randomized studies, and the Risk Of Bias In Non-randomized Studies of Interventions 1 tool to assess the risk of bias across the observational studies across all risk domains [21,22]. The results of the risk assessment were represented as a “traffic lights” plot, and the individual risk domains were summarized via a “summary plot.”

Standardization of Nonpoolable Data

For the studies that reported continuous outcomes as means and corresponding confidence intervals (CIs), risk ratios (RRs) were derived using established statistical methods. Specifically, the standard error (SE) was calculated from the CI bounds using the formula:

$\textrm{SE} = \frac{\text{Upper CI} - \text{Lower CI}}{2 \times t_{\alpha/2,\mathrm{df}}}$

where SE represents the standard error, Upper CI indicates the upper confidence interval bound, Lower CI represents the lower confidence interval bound, and tα/2,df represents the critical value from the Student’s t-distribution for the selected confidence level and degrees of freedom $(\textrm{df} = \textrm{n} {-} 1)$. The RR was then obtained by multiplying the SE by the square root of the sample size. This approach is consistent with guidance from the Cochrane Handbook for Systematic Reviews of Interventions (version 6.4, Section 6.5.2.3) for converting summary statistics when raw data are unavailable [23].

Statistical Analysis

All the data in the investigation were available as dichotomous variables. The pretest and posttest interventions were extracted for continuous outcomes. Due to a lack of paired t-test data, "n/N" (frequency of events divided by total number of events) was utilized independently in crossover analyses. In the meta-analysis, many data points were employed to analyze the heterogeneity of the publications included. The effect size varied among studies, as indicated by the tau square (τ^2^), indicating within-study variance. Degrees of freedom (df) denote the number of independent comparisons needed to determine the pooled effect size. The chi-square (χ^2^) test was used to evaluate if there were significant variations in effect sizes between studies compared to random chance. If the chi-square value was substantial, heterogeneity was considered significant. The I^2^ statistic was used to determine the proportion of total variance attributable to heterogeneity rather than random variation. Since the statistical heterogeneity was assessed using the I² statistic, values more than 50% indicated significant heterogeneity. Elevated scores indicated greater variety and variance among research findings. The analysis was represented by "forest plots" with a null-effect line in the middle axis and a "diamond" reflecting the combined effect of various investigations. RevMan (version 5.4.1; The Cochrane Collaboration, London, UK) was used to analyze the 10 primary studies [24].

Results

Characteristics of the Included Studies

This systematic review and meta-analysis included 10 distinct studies evaluating the timing of invasive strategies in patients with NSTEMI or NSTE-ACS. The nature of the included studies varied significantly, encompassing randomized trials, retrospective single-center studies, observational analyses of nationwide registries, and collaborative analyses of randomized trials. The total number of patients included in individual studies ranged widely, from 54 to 363,500 diabetic patients. The studies were conducted across various geographical locations, including Japan, Italy, Israel, Serbia, the United States, South Korea, and Europe. The populations studied focused predominantly on NSTEMI patients, often with high-risk features, such as those with severe left ventricular ejection fraction (<35%) or chronic kidney disease (CKD). One study focused exclusively on diabetic patients with NSTEMI. The primary outcomes reported across these studies varied, though most centered on mortality, MI, or a composite thereof. Specifically, major adverse cardiovascular events (MACE), defined as the composite of all-cause death, nonfatal MI, and readmission for heart failure, were explicitly defined as the primary endpoint across a majority of the included studies. The intervention timing varied significantly: immediate (six hours or less than two hours) and early (24 hours or two days) compared against delayed, selective, or initial conservative strategies (defined as >24 hours, 24-48 hours, 7-72 hours, 2-72 hours, or greater than three days). Percutaneous coronary intervention (PCI) was the main revascularization procedure applied and studied for timing in eight studies, while one study examined the timing of CABG. A summary of the interventions and the outcomes reported across the included studies is mentioned in Table 3.

**Table 3:** Characteristics of included studies IQR: interquartile range; PCI: percutaneous coronary intervention; MACE: major adverse cardiovascular events; MI: myocardial infarction; AMI: acute myocardial infarction; CA: coronary angiography; MCE: myocardial contrast echocardiography; CDL: contrast defect length; MBG: myocardial blush grade; CK-MB: creatine kinase-myocardial band; TnT: troponin T; AUC: area under curve; LVEF: left ventricular ejection fraction; LVEDV: left ventricular end-diastolic volume; LVESV: left ventricular end-systolic volume; CV: cardiovascular; HR: hazard ratio; RI: routine invasive; SI: selective invasive; CABG: coronary artery bypass grafting; AKI: acute kidney injury; GIH: gastrointestinal hemorrhage; LOS: length of stay; D2BT: door-to-balloon time; MACCE: major adverse cardiac and cerebrovascular events; NSTEMI: non-ST-elevation myocardial infarction; CKD: chronic kidney disease

S. no.	Study	Mean age	Male:female	Procedure used	Time periods and intervals	Primary outcomes	Secondary outcomes
1	Ota et al. [25]	Median: 73.0 years (66.0-80.0 IQR). Early PCI: 73.0 (65.0-79.0 IQR) years; delayed PCI: 76.0 (69.0-81.0 IQR) years	75.5% male (616/816). Early PCI: 79.4% male; delayed PCI: 70.8% male (p = 0.005)	PCI	Early PCI: PCI performed within 24 hours of presentation. Delayed PCI: PCI was performed 24 hours after presentation. Median follow-up: 856 days	MACE: composite of all-cause death, nonfatal MI, and readmission for heart failure	All-cause death, readmission for heart failure, nonfatal AMI
2	Sciahbasi et al. [26]	Immediate PCI: 58.8 ± 9.4 years; early PCI: 59.7 ± 9.8 years	Immediate PCI: 81.5% male (22/27); early PCI: 88.9% male (24/27)	PCI (all procedures performed via transradial approach). Glycoprotein IIb/IIIa inhibitor (eptifibatide) was used in both arms	Immediate: 6 hours after hospital admission. Early: 7-72 hours after hospital admission. Median time from admission to CA: 5 hours (immediate) vs. 24 hours (early) (p < 0.0001). Follow-up: 1 year	Microvascular damage (MCE/CDL), myocardial perfusion (MBG), and infarct size (CK-MB/TnT peak levels)	AUC of CK-MB and TnT. Echocardiographic parameters (LVEF, LVEDV, LVESV) at predischarge and 1 year
3	Mahmoud et al. [27]	Matched cohort: 67 ± 11 years (early invasive) vs. 68 ± 13 years (conservative). overall: 68.1 ± 12.6 years	Overall: 42.9% female. Matched cohort: 42% female in both groups	Coronary angiography, PCI, or CABG	Early invasive: within 48 hours of admission (day 0 or 1). Conservative: PCI/CABG performed >48 hours, or none performed	Incidence of in-hospital mortality	LOS stay, total hospital charges
4	Damman et al. [28]	Median: 65 years (56-72 IQR). Early Angio: 63 (55-71 IQR) years; delayed Angio: 66 (57-72 IQR) years (p < 0.001)	68.6% male (1451/2116). No significant difference between groups (p = 0.67)	Risk factors included history of MI (26%), previous PCI (7%), previous CABG (3%), current smoking (32%), hypercholesterolemia (25%)	5-year composite outcome of CV death or MI	No difference observed in 5-year CV death or MI in unadjusted (HR: 1.06, p = 0.61) or adjusted (HR: 0.93, p = 0.54) Cox models	No differences in CV death (p = 0.94) or MI (p = 0.37). Major bleeding was comparable (3.3% early vs. 3.1% delayed, p = 0.86)
5	Henderson et al. [29]	Mean age: 62 years	38% female	Coronary arteriography, PCI, or CABG. RI group had 55% revascularization during index hospitalization vs. 10% in SI group	10-year all-cause mortality	No difference in all-cause mortality at 10 years (RI: 25.1% vs. SI: 25.4%, p = 0.94). The mortality advantage seen at 5 years was attenuated during later follow-up (HR 0-5 years: 0.76; HR 5-10 years: 1.28; p = 0.006 for interaction)	No difference in cardiovascular mortality (RI: 15.1% vs. SI: 16.1%, p = 0.65). No evidence for interaction between risk score and treatment effect on mortality at 10 years
6	Milosevic et al. [30]	Median: immediate: 60.5 (52-69 IQR) years; delayed: 63.0 (55-71 IQR) years	Female: 29.6% (immediate) vs. 34.2% (delayed)	Invasive intervention (coronary angiography and PCI/CABG). PCI rate higher in immediate group (78.4% vs. 65.0%)	Immediate-intervention: 2 hours after randomization. Delayed-intervention: 2-72 hours after randomization	Composite of death or new MI at 30-day follow-up	Composite of death, new MI, or recurrent ischemia at 30 days and 1 year. Death or new MI at 1 year. Major bleeding
7	Park et al. [31]	Ranged from 64.6 (≤24 hours) to 66.0 (>120 hours) years	Ranged from 74.7% male (≤24 hours) to 69.6% male (>120 hours)	CABG	Timing of CABG: 24 hours, 24-72 hours, 72-120 hours, and >120 hours from admission. Data years 2016-2020	In-hospital mortality	Perioperative complications (e.g., AKI, GIH, non-home discharge), LOS, total hospital cost
8	Arora et al. [32]	Early PCI: 59 ± 0.3 years; late PCI: 61 ± 0.2 years (p = 0.01)	Early PCI: 72% male; late PCI: 66% male (p = 0.0009)	PCI	Early PCI: <24 hours of the event onset. Late PCI: ≥24 hours of the event onset. Follow-up: 28-day and 1-year mortality	28-day and 1-year mortality	Mortality stratified by risk groups and presentation timing
9	Shin et al. [33]	Mean: 69.5 ± 11.8 years. Age similar between groups	67.4% male	PCI	Early invasive: PCI within 24 hours (D2BT 24 hours). Selective invasive: PCI over 24 hours (D2BT >24 hours). Follow-up: 12 months	MACCE at 12 months (composite of all-cause death, nonfatal MI, repeat revascularization, and stroke)	In-hospital death, cardiogenic shock after PCI, 30-day MACCE, and maximum troponin I level
10	Sharon et al. [34]	Median: 66 years (58-74 IQR). Early invasive: 63 ± 12 years; delayed invasive: 68 ± 12 years (p < 0.001)	77% male (2,711/3,529). Early: 79% male; delayed: 74% male (p < 0.001)	Coronary angiography (followed by intervention)	Early invasive: coronary angiography 24 hours from NSTEMI diagnosis. Delayed invasive: coronary angiography >24 hours. Median follow-up: 4 years	All-cause mortality (long-term survival)	Short-term (30-day) mortality. Mortality stratified by CKD stages

Findings of the Primary Outcomes

Regarding the impact of early intervention on the primary outcomes, five of 10 (50%) studies demonstrated a significant benefit favoring early/immediate strategies, while four of 10 (40%) studies found no benefit in adjusted/long-term analyses. Additionally, one of 10 (10%) studies concluded that the early invasive strategy was associated with increased risk of adverse events. Milosevic et al. [30] found that immediate intervention (less than two hours) was associated with a lower rate of the primary endpoint of death or new MI at 30-day follow-up compared with delayed intervention (2-72 hours). This difference persisted at one year (6.8% vs. 18.8%; hazard ratio (HR) = 0.34). Arora et al. [32] reported that early PCI (<24 hours of event onset) was associated with a 58% reduced 28-day mortality for the entire population (OR = 0.42) and a 57% reduced 28-day mortality for high-risk patients (OR = 0.43) compared with late PCI (>24 hours). Mahmoud et al. [27] found that an early invasive strategy (within 48 hours) was associated with lower in-hospital mortality (2.2% vs. 3.8%; OR = 0.57) compared with an initial conservative strategy in propensity-matched diabetic NSTE-ACS patients.

Further, Sharon et al. [34] reported that the early invasive strategy (24 hours to angiography) was associated with a significant 30% lower mortality (HR = 0.70) compared with the delayed invasive strategy (>24 hours) after adjusting for baseline differences using inverse probability treatment weighting. Conversely, Ota et al. [25] reported that, while MACE was more frequently observed in the delayed PCI group (40.8% vs. 28.5%) in unadjusted analysis, multivariate Cox hazard analysis revealed that early PCI was not associated with MACE. Similarly, Damman et al. [28], analyzing patients randomized to a routine invasive strategy, found no difference in the five-year composite outcome of cardiovascular death or MI in either unadjusted (HR = 1.06) or adjusted models (HR = 0.93) when comparing early angiography (within two days) vs. delayed angiography (within three to five days).

Secondary Outcomes

Several other outcomes were reported concerning mortality, ischemic events, and procedural complications. Mortality findings beyond the primary composite endpoint showed that the mortality advantage of a routine early invasive strategy observed at five years in the RITA-3 trial was attenuated during later follow-up, resulting in no difference in all-cause mortality at 10 years [29]. The survival benefit associated with an early invasive strategy was significantly modified by CKD [34], with the benefit being limited to patients with estimated glomerular filtration rate ≥45 mL/min/1.73 m³. Regarding component outcomes of MACE, Ota et al. [25] found that readmission for heart failure was significantly more frequent in the delayed PCI group (21.9% vs. 8.3%), but there was no significant difference in nonfatal AMI. New MI events were significantly more frequent in the delayed intervention group compared with the immediate intervention group in the precatheterization period (10 MIs vs. 0 MIs) [30].

Furthermore, infarct size was addressed by Sciahbasi et al. [26], who found that immediate PCI (six hours) was associated with a significant reduction in myonecrosis markers (creatine kinase-myocardial band peak 26 ± 26 vs. 69 ± 79 ng/mL; troponin T peak 0.84 ± 1.2 vs. 1.8 ± 2.1 ng/mL) compared with PCI within 7-72 hours. Recurrent ischemia was reported to be lower in the immediate-intervention group compared with the delayed-intervention group at 30 days (3.7% vs. 15.5%) [30]. Similarly, Damman et al. found major bleeding was comparable between early and delayed angiography groups (3.3% vs. 3.1%) [28]. Conversely, Shin et al. reported that the early invasive group had a higher incidence of in-hospital death (9.4% vs. 3.3%) and cardiogenic shock after PCI (11.5% vs. 4.6%) [33]. Table 4 summarizes the findings of the individual studies.

**Table 4:** Results of the systematic review NIS: National Inpatient Sample; NSTE-ACS: non-ST-segment elevation acute coronary syndrome; HT: hypertension; CAD: coronary artery disease; UA: unstable angina; NSTEMI: non-ST-elevation myocardial infarction; PCI: percutaneous coronary intervention; STEMI: ST-elevation myocardial infarction; AMI: acute myocardial infarction; MI: myocardial infarction; CA: coronary angiography; PCI: percutaneous coronary intervention; CAD: coronary artery disease; FRISC II: Fragmin and Fast Revascularization During Instability in Coronary Artery Disease; ICTUS: Invasive Versus Conservative Treatment in Unstable Coronary Syndromes; RI: routine invasive; SI: selective invasive; CK: creatine kinase; RITA-3: Intervention Versus Conservative Treatment Strategy in Patients With Unstable Angina or Non-ST Elevation Myocardial Infarction; RIDDLE-NSTEMI: Randomized study of Immediate versus Delayed Invasive Intervention in patients with Non ST-segment Elevation Myocardial Infarction; NIS: National Inpatient Sample; VF: ventricular fibrillation; ARIC: atherosclerosis risk in communities; TIMI: Thrombolysis In Myocardial Infarction; KAMIR-V: Korean Acute Myocardial Infarction Registry V; LV: left ventricular; LVEF: left ventricular ejection fraction; GRACE: Global Registry of Acute Coronary Events; IPTW: inverse probability treatment weighting; CKD: chronic kidney disease; eGFR: estimated glomerular filtration rate; MCS: mechanical circulatory support; ULN: upper limit of normal

S. no.	Study	Study location	Study design	Sample size	Population characteristics	Intervention	Main findings	Conclusion
1	Ota et al. [25]	Saitama Medical Center, Jichi Medical University, Japan	Single-center, retrospective study	816 patients with NSTEMI (early PCI: n = 446, delayed PCI: n = 370)	Patients with NSTEMI. Exclusion of STEMI, PCI not performed for culprit lesion, in-hospital onset AMI, or multiple NSTEMI episodes	Comparison of early PCI vs. delayed PCI for the culprit lesion	MACE was more frequently observed in the delayed PCI group (unadjusted). However, after multivariate adjustment, early PCI was not associated with MACE	The timing of PCI should be determined based on the patient’s clinical status and comorbidities, as early or delayed PCI may not be important in hemodynamically stable patients with NSTEMI
2	Sciahbasi et al. [26]	Rome, Italy	Randomized study	54 consecutive patients with first episode of NSTEMI (immediate PCI: n = 27, early PCI: n = 27)	Patients with their first episode of acute NSTEMI. Exclusion criteria included previous MI or revascularization, severe hemodynamic impairment, or cardiogenic shock	Comparison of immediate invasive strategy (CA and PCI at 6 hours) vs. early invasive strategy (CA and PCI 7-72 hours)	Immediate PCI is associated with less increase in myonecrosis markers (infarct size) compared with PCI within 72 hours, despite similar myocardial perfusion	Immediate PCI strategy associated with lower extent of infarct size but has no effect on myocardial perfusion in NSTEMI patients treated with eptifibatide
3	Mahmoud et al. [27]	United States (NIS database)	Retrospective, propensity score matched analysis	Total: 363,500 diabetics with NSTE-ACS. Matched: 21,681 patients in each arm	Diabetics with NSTE-ACS (NSTEMI or unstable angina). High comorbidities (HT 82.8%, CAD 77.9% overall)	Comparison of early invasive strategy (CA ± revascularization within 48 hours of admission) vs. initial conservative strategy (remaining patients)	Early invasive strategy associated with increased in-hospital survival in diabetic NSTE-ACS patients. This benefit was superior in NSTEMI compared with UA	An early invasive strategy associated with improved in-hospital survival in diabetic patients, especially those with high-risk features (NSTEMI or cardiogenic shock). Initial conservative strategy may be safer for diabetics with UA
4	Damman et al. [28]	Amsterdam, The Netherlands; Uppsala, Sweden; Edinburgh, and London, UK	Collaborative analysis of individual patient data from the routine invasive arms of FRISC II, ICTUS, and RITA-3 trials	2,116 patients undergoing angiography (early Angio: n = 975, delayed Angio: n = 1,141)	Patients with non-ST-segment elevation acute coronary syndrome (nSTE-ACS) randomized to a routine invasive strategy	Comparison of early angiography vs. delayed angiography in patients undergoing routine invasive management	In patients presenting with nSTE-ACS and randomized to a routine invasive strategy, early angiography (within 48 hours) does not reduce the incidence of 5-year death or MI compared with delayed angiography (48-120 hours)	-
5	Henderson et al. [29]	United Kingdom	10-year follow-up of the RITA-3 randomized trial	1,810 patients with NSTE-ACS randomized. (RI: n = 895, SI: n = 915)	Patients with NSTE-ACS (unstable angina or NSTEMI). Exclusion if routine coronary arteriography planned within 72 hours or if CK levels elevated to twice ULN before randomization	Comparison of routine early invasive strategy vs. selective invasive strategy	For most patients with NSTE-ACS eligible for either strategy, neither confers a prognostic advantage over 10 years. Conservative treatment remains a reasonable option for lower risk patients	-
6	Milosevic et al. [30]	Belgrade, Serbia	Randomized, parallel-group, open-label, single-center trial (RIDDLE-NSTEMI Study)	323 NSTEMI patients (immediate-intervention: n = 162, delayed-intervention: n = 161)	Initially stabilized NSTEMI patients only. Exclusion: persistent ST-segment elevation, hemodynamic instability, cardiogenic shock, or refractory angina on admission	Comparison of immediate intervention vs. delayed intervention	Immediate invasive strategy associated with lower rates of death or new MI at 30 days and 1 year	Immediate invasive strategy (<2 hours) in NSTEMI patients is associated with lower rates of death or new MI compared with delayed invasive strategy (2-72 hours), mainly due to a decrease in the risk of new MI in the precatheterization period
7	Park et al. [31]	United States (NIS database)	Retrospective analysis	147,170 NSTEMI hospitalizations where CABG was performed	NSTEMI patients requiring CABG. High-risk features (shock, VF, cardiac arrest, PCI/MCS before CABG) were excluded to create a more homogeneous population	Comparison of outcomes according to timing of CABG surgery	The optimal timing of CABG has not been determined, but contemporary data suggest similar in-hospital mortality across time intervals in this selected stable population. Increased complications/costs found with delays	The timing of a CABG approach does not result in a significant overall mortality difference for NSTEMI patients, but delaying CABG beyond 72 hours leads to higher complications, costs, and LOS
8	Arora et al. [32]	Four US communities (ARIC surveillance study)	Observational study/retrospective analysis	6,746 patients hospitalized with NSTEMI who underwent PCI. (early PCI: n = 2,376, late PCI: n = 4,370)	Hospitalized NSTEMI patients amenable to PCI. Patients stratified by TIMI risk score. High prevalence of ST-segment deviation (74% early)	Comparison of early PCI vs. late PCI	Early PCI is associated with improved 28-day survival in high-risk NSTEMI patients undergoing revascularization	In-hospitalized NSTEMI patients at high risk of clinical events and amenable to PCI, early intervention is associated with improved 28-day survival
9	Shin et al. [33]	South Korea (KAMIR-V registry)	Observational prospective study (multicenter, nationwide)	386 NSTEMI patients with severe LV dysfunction (LVEF 35%)	NSTEMI patients with severe left ventricular dysfunction (LVEF = 35%). Exclusion of cardiogenic shock. High mean GRACE score (169.7 ± 47.4)	Comparison of early invasive group vs. selective invasive group	The early invasive strategy did not improve clinical outcomes and was associated with increased risks of in-hospital death and cardiogenic shock	Among NSTEMI patients with severe LV dysfunction (LVEF = 35%), an early invasive strategy within 24 hours did not improve their clinical outcomes, suggesting an individualized approach is beneficial
10	Sharon et al. [34]	Sheba Medical Center, Israel	Retrospective cohort study/analysis using IPTW	3,529 invasively treated NSTEMI patients (early invasive: n = 1,837; delayed invasive: n = 1,692)	Invasively treated NSTEMI patients, stratified by CKD status (eGFR)	Evaluation of the association between early invasive strategy vs. delayed invasive strategy and long-term survival	The association between early invasive strategy and long-term survival is significantly modified by CKD. The survival benefit of the early invasive strategy is limited to patients with eGFR = 45 mL/min/1.73 m²	Among NSTEMI patients, the association of early invasive strategy with long-term survival is modified by CKD and was not observed in patients with eGFR < 45 mL/min/1.73 m²

Results of the quality assessment

**Figure 2:**
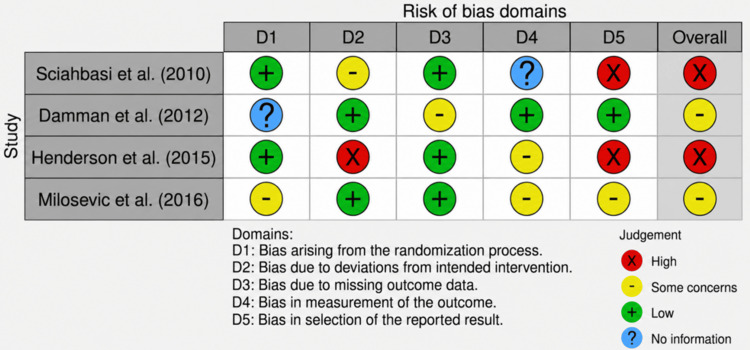
Traffic lights for randomized studies via ROBv2 tool ROBv2: Risk of Bias v2 Source: [26,28-30]

**Figure 3:**
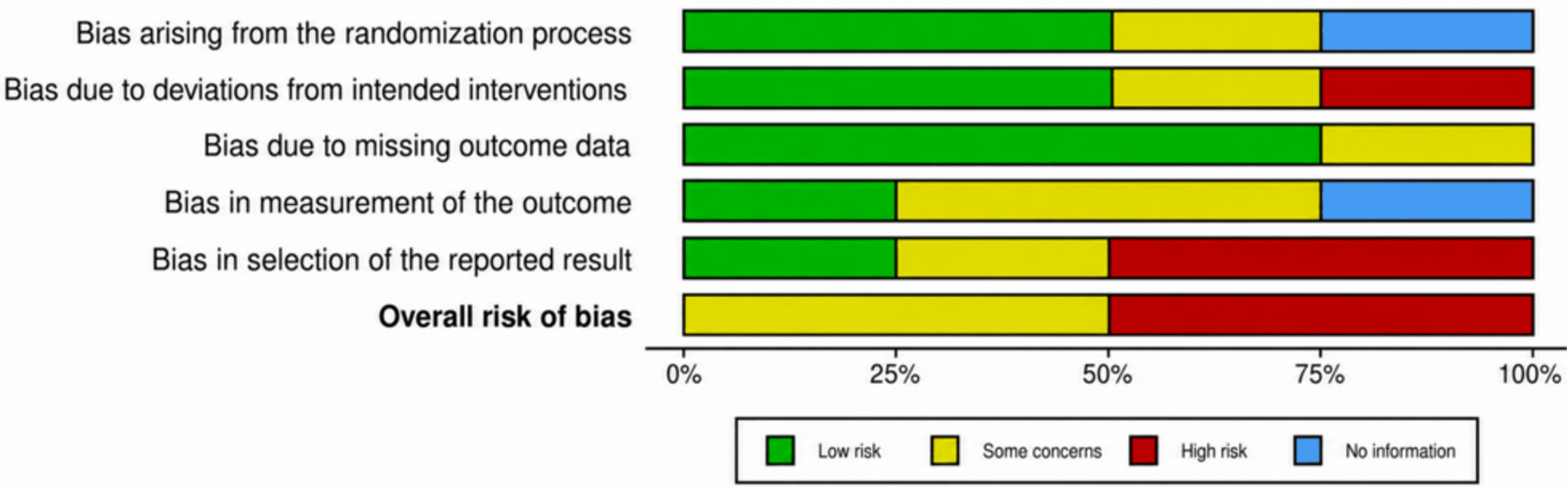
Summary plot for individual risk for randomized studies Source: [26,28-30]

**Figure 4:**
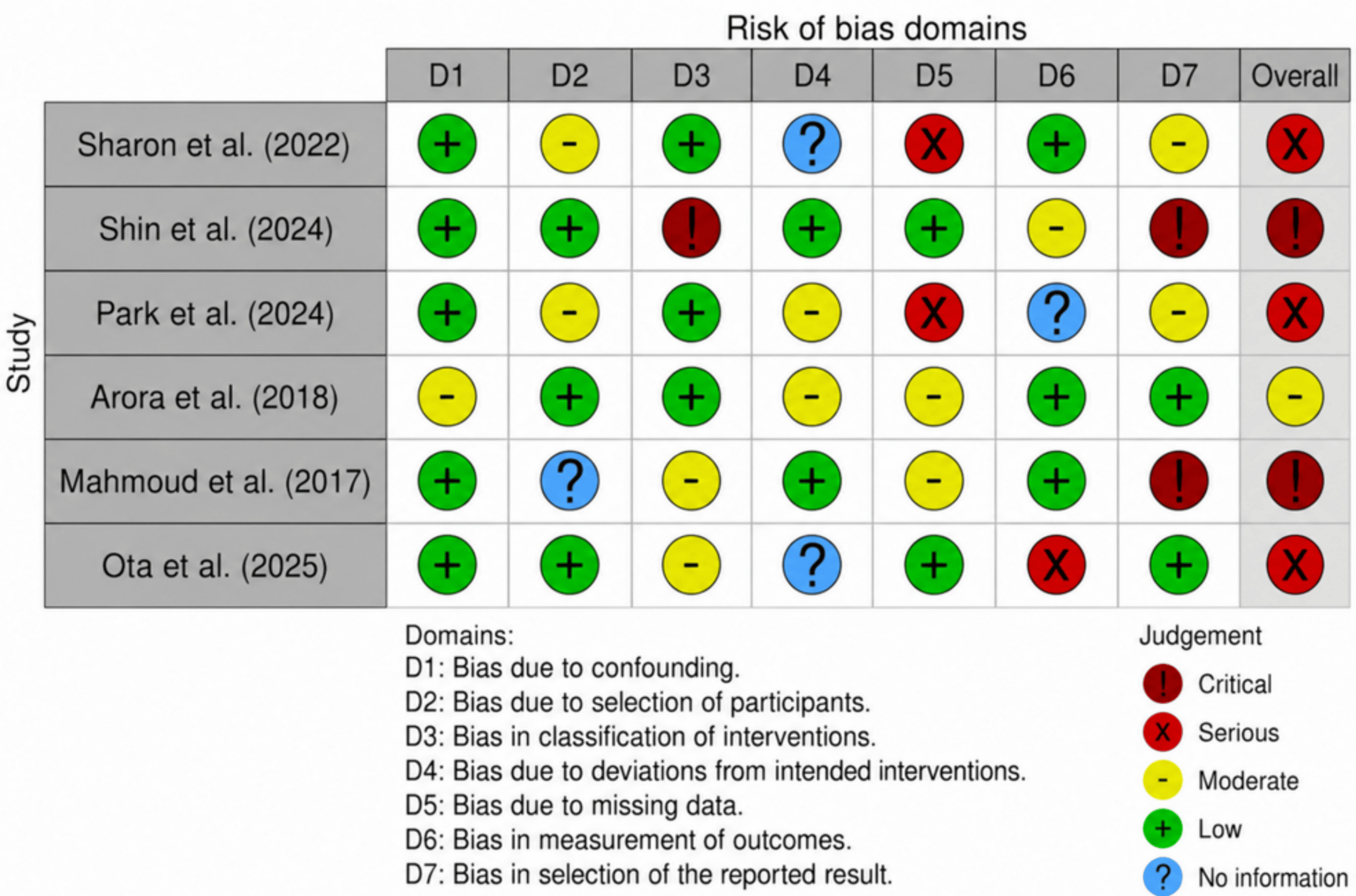
Traffic lights plot for nonrandomized studies Source: [25,27,31-34]

**Figure 5:**
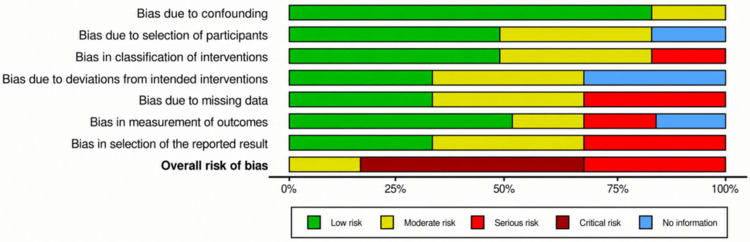
Summary plot for individual domains in nonrandomized studies Source: [25,27,31-34]

The results of the bias assessment are shown in the “traffic lights” plots, and the individual risk in each domain was further illustrated by “summary plots” for randomized and nonrandomized studies (Figures 2-5).

Meta-analysis

Primary Outcomes (MACE)

**Figure 6:**
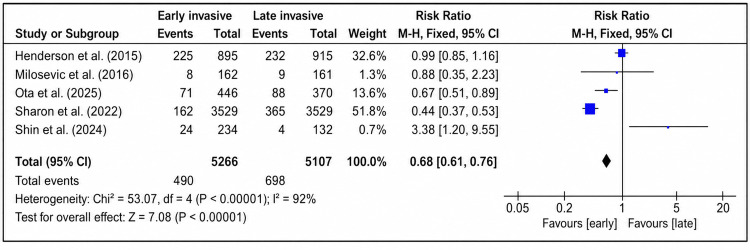
Forest plot for all-cause death in early vs. late intervention groups M-H: Mantel-Haenszel; CI: confidence interval Source: [25,29,30,33,34]

All-cause death: A total of 5 studies reported quantitative data on all-cause death, comparing early and late invasive study groups. As evident from the plot, four of five (80%) studies showed a positive association between the studied variables. The overall risk of all-cause death favored early intervention and induced a protective effect in patients undergoing early/immediate management for NSTEMI. On the other hand, one of five (20%) studies showed a negative association and reported a reduction in all-cause mortality with the late/delayed intervention cohort. The overall estimate is calculated in the form of RR, given an RR = 0.68 (95% CI: 0.61-0.76), with a statistically significant p value (p < 0.00001). The heterogeneity in the analysis was found to be significant (I² > 50), which was contributed to by different diagnostic criteria, study populations, and measured variables across the study group. The forest plot for the analysis is provided in Figure 6.

**Figure 7:**
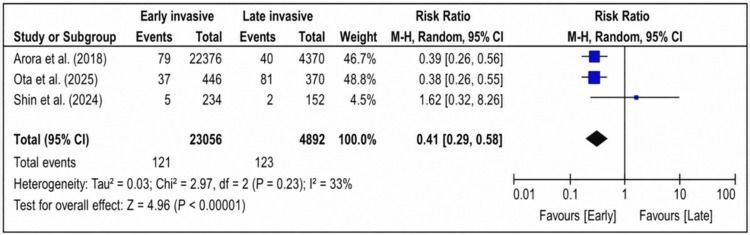
Forest plot for rehospitalization due to heart failure M-H: Mantel-Haenszel; CI: confidence interval Source: [25,32,33]

Rehospitalization due to heart failure: The second outcome variable under MACE was readmission or rehospitalization due to secondary heart failure in early vs. delayed intervention groups. Only three studies provided the outcome data, out of which two of three (66%) studies reported a reduced incidence of rehospitalization with early invasive strategies (within 6 or <24 hours) (PCI or CABG) compared with delayed intervention (>24 hours or three to five days). The overall effect size was calculated to be RR = 0.41 (95% CI: 0.29-0.58). The analysis was significant (p < 0.0001), with negligible heterogeneity (I² = 33%). We inferred that early/immediate revascularization was associated with a decreased risk of readmission within 28 days and at one-year follow-up. The forest plot for the analysis is mentioned in Figure 7.

**Figure 8:**
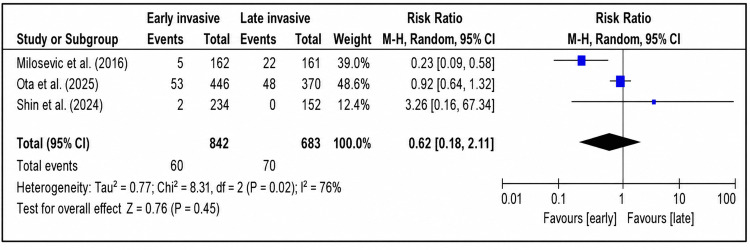
Forest plot for nonfatal MI for early vs. late intervention groups M-H: Mantel-Haenszel; CI: confidence interval; MI: myocardial infarction Source: [25,30,33]

Non-fatal AMI: The third outcome for the study was the overall incidence of nonfatal AMI in patients who underwent early revascularization vs. those who underwent a selective invasive strategy/conservation strategy/or delayed strategy. However, the results of the analysis were found to be inconclusive. Of the three studies, one reported a positive association, one a negative association with early intervention, and one a small but insignificant effect (no effect) when the two study groups were compared. The population samples were comparable. The effect size was as follows: RR = 0.62 (95% CI: 0.18-2.11) and p value = 0.45, which was statistically insignificant. Heterogeneity was also significant, with a large CI, reflecting a lack of reported evidence and variable criteria for measuring the overall effect across individual studies (Figure 8).

Secondary Outcome

**Figure 9:**
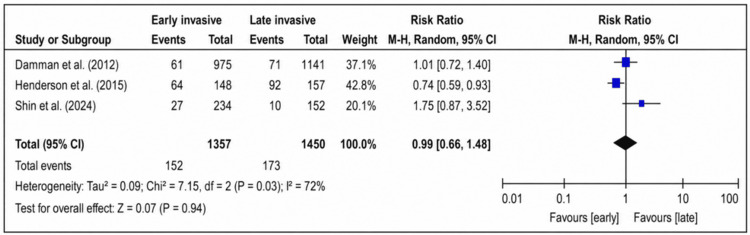
Forest plot for CV death at five years M-H: Mantel-Haenszel; CI: confidence interval; CV: cardiovascular Source: [28,29,33]

CV death (5-year): The relative incidence of cardiovascular death was measured in only a few studies. CV death across the studies showed a small but insignificant difference at one year, but the regression analysis at three, five, and 10 years revealed no association. The analysis measured the incidence at five years and demonstrated a small but statistically insignificant effect. The RR was 0.99 (95% CI = 0.66-1.48), p=0.94, and high heterogeneity (I² = 72%) as shown in Figure 9.

Discussion

Interpretation of the Findings

This systematic review and meta-analysis evaluated the effectiveness of early vs. delayed invasive revascularization strategies in patients with NTEMI. The primary findings demonstrate that early invasive revascularization strategies (defined as intervention within 2-24 hours) are associated with a significant reduction in MACE compared with delayed or selective invasive approaches. Specifically, our meta-analysis revealed a significant association favoring early intervention for the composite endpoint of all-cause death and rehospitalization due to heart failure outcomes. However, no significant benefit was observed for nonfatal AMI as an individual endpoint, suggesting that the protective effects of early intervention may be primarily driven by reductions in mortality and heart failure-related outcomes rather than prevention of subsequent MI. The heterogeneity observed across studies reflects the complexity of NSTEMI management and the diverse patient populations included. Studies included patients with varying risk profiles, from high-risk populations with severe left ventricular dysfunction (LVEF ≤ 35%) to specific subgroups such as diabetic patients and those with CKD [33,34]. This diversity strengthens the generalizability of our findings while highlighting the importance of individualized treatment approaches.

Comparison With Existing Literature

Our findings align with and extend several previous systematic reviews and meta-analyses on invasive timing strategies in NSTEMI, while also revealing important differences warranting discussion. Zhang et al. [35] conducted a systematic review comparing early- and delayed-invasive strategies in NSTE-ACS, focusing on short- to medium-term outcomes. Their analysis, similar to ours, found heterogeneity in trial timing definitions but concluded that early invasive strategies did not consistently reduce all-cause death or MI across all patient populations. However, they noted consistent reductions in recurrent or refractory ischemia with earlier intervention. Our analysis extends these findings by demonstrating a significant reduction in MACE when examining a broader composite endpoint that includes heart failure rehospitalization, suggesting that previous analyses may have underestimated the clinical benefits of early intervention by focusing primarily on death and MI as separate endpoints.

Velagapudi et al. specifically examined ultraearly intervention (less than two hours) vs. later strategies in NSTEMI patients, analyzing three RCTs totaling 1,075 patients [36]. Their meta-analysis found no statistically significant differences in all-cause mortality, MI, or major bleeding between ultraearly and delayed approaches. While this appears to contrast with our findings, it is important to note that their analysis was limited to only three trials and focused on very immediate intervention timing. Our broader inclusion criteria and larger patient population provide more robust evidence supporting the benefits of early intervention, particularly when examining composite endpoints that better reflect overall cardiovascular outcomes. A systematic review by Reaño et al. focused specifically on elderly patients (≥65 years) comparing invasive vs. conservative strategies [37]. They found mortality and MI benefits in elderly patients but noted substantial between-study heterogeneity. Our analysis includes several studies with elderly populations, and our findings support the benefits of early invasive strategies across age groups, though we acknowledge the importance of individualized risk assessment in older patients.

Recent narrative reviews and guideline-focused syntheses have emphasized selective application of early invasive timing, prioritizing very high-risk patients (GRACE score >140) for early angiography while allowing planned delays for lower risk patients [38,39]. Our findings support this individualized approach and provide stronger evidence of early intervention benefits than previously reported. The consistent MACE reduction observed across our diverse study population suggests that the benefits of early intervention may be more broadly applicable than suggested by previous analyses that focused primarily on hard endpoints of death and MI.

Clinical Implications

The findings of this meta-analysis have several important clinical implications for the management of NSTEMI patients. The demonstrated reduction in MACE with early invasive strategies supports current guideline recommendations favoring early intervention in high-risk NSTEMI patients and extends the evidence base to suggest broader applicability of this approach. The significant reduction in composite MACE outcomes, driven primarily by decreases in all-cause mortality and heart failure rehospitalization, suggests that early intervention may prevent the cascade of complications that can follow delayed revascularization [40]. This is particularly relevant given the increasing recognition of heart failure as a major long-term consequence of MI. The lack of benefit for nonfatal MI as an individual endpoint may reflect the complex pathophysiology of recurrent ischemic events, which may be influenced by factors beyond the timing of initial intervention [41]. For clinical practice, these findings support a strategy of early invasive intervention within 24 hours of presentation for most NSTEMI patients, with consideration of even more immediate intervention (≤2 hours) for the highest risk patients. However, the decision for early intervention should be individualized based on patient risk factors, comorbidities, and institutional capabilities [42]. The heterogeneity in patient populations studied also highlights the importance of risk stratification. While early intervention showed benefits across diverse populations, the magnitude of benefit may vary with individual patient characteristics such as age, left ventricular function, diabetes, and kidney function.

Barriers to Implementation

Several limitations must be acknowledged in interpreting these results. The varying definitions of "early" intervention (ranging from ≤2 to ≤24 hours) and "delayed" intervention across studies may have influenced the observed treatment effects [43]. Practical barriers to implementation of early invasive strategies include resource availability, particularly in healthcare systems with limited cardiac catheterization laboratory capacity or 24-hour interventional cardiology coverage [44]. The requirement for immediate availability of interventional cardiologists and supporting staff may limit the feasibility of very early intervention in some settings. Patient-related factors may also present barriers to early intervention, including comorbidities that increase procedural risk, patient preferences, and contraindications to anticoagulation or antiplatelet therapy [45]. The optimal timing strategy may need to be individualized based on these factors.

Strengths and Limitations of the Study

The strengths of this systematic review and meta-analysis include the comprehensive search strategy, inclusion of diverse study designs and patient populations, and focus on clinically relevant composite endpoints. The inclusion of both RCTs and observational studies provides a broader evidence base than previous analyses limited to randomized trials alone. The large total patient population studied (ranging from individual studies of 54 patients to registry studies of over 363,000 patients) enhances the statistical power and generalizability of the findings. The international scope of the included studies, spanning multiple continents and healthcare systems, further supports the external validity of the results.

However, several limitations must be acknowledged. The heterogeneity in study designs, outcome definitions, and patient populations may limit the precision of pooled estimates and introduce potential bias. The observational nature of several included studies may be subject to confounding by indication, where sicker patients may have been selected for different timing strategies. Publication bias remains a potential concern, as studies demonstrating significant benefits may be more likely to be published than those showing neutral results. The varying follow-up periods across studies (from 28 days to 10 years) may also influence the interpretation of long-term outcomes.

## Conclusions

This systematic review and meta-analysis provides robust evidence supporting early invasive revascularization strategies in NSTEMI patients. The significant reduction in MACE outcomes, driven primarily by decreases in all-cause mortality and heart failure rehospitalization, supports current guideline recommendations while extending the evidence base for early intervention benefits. The findings suggest that early invasive intervention within 24 hours of presentation should be considered the standard of care for most NSTEMI patients, with potential benefits of even more immediate intervention in the highest risk patients. However, the decision for early intervention should be individualized based on patient risk factors, comorbidities, and institutional capabilities. Future research should focus on identifying optimal timing strategies for specific patient subgroups, developing risk-stratification tools to guide timing decisions, and addressing implementation barriers across diverse healthcare settings. The consistent benefits observed across diverse patient populations and healthcare systems suggest that early invasive strategies represent an important opportunity to improve outcomes for NSTEMI patients worldwide.
